# Enhancing blood culture volume with ultrathin-wall cannula devices

**DOI:** 10.1128/jcm.00208-25

**Published:** 2025-09-05

**Authors:** Grant Johnson, Valentin Parvu, Stephane Beauchamp, Mike Cuttler, Arek Zubrzycki, Lauren Cooper

**Affiliations:** 1 Lakeridge Health 6253, Oshawa, Ontario, Canada; 2 Becton, Dickinson, and Company, Diagnostic Solutions 157841 , Sparks, Maryland, USA; 3 Becton, Dickinson and Company, Life Sciences 103910 , Mississauga, Ontario, Canada; Children's Hospital Los Angeles, Los Angeles, California, USA

**Keywords:** blood culture, blood volume, diagnostics, UltraTouch, BACTEC, cannula

## Abstract

Suboptimal blood volume collection negatively affects pathogen recovery and time to pathogen detection in clinical blood culture samples. This study evaluated the performance of the ultrathin-wall cannula device of the BD Vacutainer UltraTouch Push Button Blood Collection Set (UltraTouch; BD Life Sciences, Franklin Lakes, NJ) in increasing the blood volume collected compared to regular cannulas. In this retrospective study, the volume of blood collected in negative BD BACTEC Plus Aerobic/F Culture Vials prior to and following blind conversion to UltraTouch was measured from blood background metabolic activity signal acquired during the initial incubation period in the BD BACTEC FX instrument using a custom software application (patent pending). Differences in average fill volume between pre- and post-conversion were evaluated overall and by month. A separate logistic regression was also performed to model the relationship between monthly average blood volume increase (mL) and sample positivity rate. Prevalence rates for identified organisms were also assessed. The blood fill volume of 13,356 pre-conversion and 119,971 post-conversion bottles was compared. The average post-conversion fill volume was significantly higher than that of pre-conversion (7.56 mL [95% CI, 7.54–7.59] vs. 5.68 mL [95% CI, 5.62–5.74], respectively). This 1.88 mL increase in collected blood volume increased sample positivity from 7.0% to 7.7% (*P* = 0.002; odds ratio [OR], 1.11; 95% CI, 1.04–1.19). This study demonstrated that using an ultrathin-wall cannula blood collection device can significantly increase blood culture volume and sample positivity, supporting improved identification of patients suffering from bloodstream infections.

**IMPORTANCE:**

Substandard blood fill volume practices in clinical settings negatively affect pathogen recovery which, in turn, may impact patients’ care. Whereas professional education regarding the importance of adhering to clinical guidelines has been helpful in partly remediating the issue, the role of blood collection set technologies in optimizing blood culture volume has not been studied as thoroughly. Our study assessed the performance of the BD Vacutainer UltraTouch Push Button Blood Collection Set, which is designed with an ultrathin-wall cannula, to improve blood culture volume.

## INTRODUCTION

Blood culture collection is an essential procedure to detect the presence of microorganisms and, subsequently, determine their antibiotic susceptibility in patients with suspected bloodstream infections (1–3). In 2021, in the USA, over 7.8 million blood culture collections were requisitioned during emergency room (ER) visits, accounting for 5.4% of all procedures performed during patients’ assessment in the ER (4). Blood cultures are also commonly performed when underlying diseases or conditions that increase the likelihood of developing sepsis are present, such as catheter-related bloodstream infections, pneumonia, and soft tissue infections (5).

Adequate bottle blood fill volume is the most important variable to ensure accurate detection of microorganisms in blood cultures (2), and it has been estimated that a 3.5% relative increase in blood culture positivity rate can be achieved for each additional 1 mL of blood volume cultured (6). However, numerous studies have reported widespread substandard blood fill volume practices (7–11). For example, Henning et al. found that from an evaluated sample of 10,328 blood culture bottles, 8,444 (81.8%) did not meet the recommended fill volume (11). Since the fill volume of blood culture bottles directly impacts the rate of pathogen recovery in positive samples (6, 8, 12, 13), it is crucial that both clinical practices and technology be aligned with the objective of attaining optimal blood fill volumes. Whereas healthcare professional education and increased laboratory feedback to healthcare providers have proven helpful in partially remediating this concern (8, 14, 15), the role of different blood collection set technologies in optimizing blood culture collection has not been studied as thoroughly (15). In this context, our study assessed the performance of an ultrathin-wall cannula blood collection device on blood fill volumes and blood culture sample positivity.

## MATERIALS AND METHODS

This retrospective study was conducted at four Lakeridge Health hospitals (Bowmanville, Oshawa, Port Perry, and Whitby) in Ontario, Canada, and aimed to compare the average blood culture fill volume and sample positivity rate before and after a conversion from the BD Vacutainer Push Button Blood Collection Set, which uses standard thin-wall cannulas (referred to herein as “regular” device), to the BD Vacutainer UltraTouch Push Button Blood Collection Set (referred herein as “UltraTouch”), which uses ultrathin-wall cannulas (both devices from BD Life Sciences, Franklin Lakes, NJ). Although the specific gages used throughout the study were not documented each time a blood draw was performed, both the UltraTouch and the regular blood collection sets were compatible with 21G, 23G, and 25G cannulas in both the pre- and post-conversion phases. The UltraTouch is a sterile, single-use, fixed-wing blood collection set intended for venipuncture and was introduced to the market in 2015. This needle was designed with ultrathin walls that provide for increased inner diameter (e.g., the inner diameter of the 25G ultrathin-wall cannula is compared to that of the 23G regular device cannula) (16). The increased inner diameter allows for faster flow, requiring up to 50% less filling time than the regular device to collect an equivalent volume of blood (17).

In our investigation, the UltraTouch was used in conjunction with the BD BACTEC Plus Aerobic/F Culture Vials. The volume of blood collected in these bottles was measured from blood background metabolic activity signal acquired during the initial incubation period in the BD BACTEC FX instrument, using a custom software application (patent pending). Positivity was defined as the number of positive bottles after subtraction of site-defined “contaminated” samples. The Synapsys software utilized with the BACTEC instrument included rules set by the laboratory to determine contaminants based on specific species of organisms and the number of bottles or sets in which they were identified. Therefore, the criteria for determining contaminants, since they are built into the Synapsys software, may differ from those in the electronic medical record (EMR) of the patients. For example, a skin commensal organism found in multiple blood culture sets might be considered a contaminant if there are no correlations with the patients’ symptoms. Conversely, bacterial growth on a prosthetic heart valve, despite negative blood culture, would indicate a bloodstream infection (BSI) in the EMR. The pre-conversion period spanned from September 2, 2018, to March 17, 2019, while the post-conversion period extended from April 22, 2019, to September 30, 2023. All sites were converted from the regular to the UltraTouch blood collection device in a blinded fashion, meaning that device users were not notified of the switch. This blind switch process was possible as no additional training was required to use the new device, with the old and new devices being almost identical to the user. Data extraction was performed at each site utilizing the BD Synapsys Informatics System connected to the BD BACTEC FX instruments. The measurement accuracy of this blood volume monitoring system (BVMS) was confirmed in a publication by Cattoir et al. in which a mean difference of −0.3 mL or −4% was found between the estimated mean volume of the BVMS and the mean weight-based volume (18).

Blood culture samples were collected from pediatric and adult populations under standard of care procedures. Whereas pediatric bottles were not measured in the study, patients from the pediatric population (<18 years of age) would have been included if their sample was collected in a BACTEC Plus aerobic bottle. To ensure data accuracy, samples collected during the washout period of March 18, 2019 to April 21, 2019, were also excluded from the data set to allow for regular device depletion at all sites.

The retrospective data used in this study were deemed exempt from requiring IRB oversight by the Advarra Institutional Review Board (IRB) [Pro00081240].

### Statistical analysis

Blood volumes calculated in the BD Synapsys System during study periods were used to compare the performance of the ultrathin-wall cannula (post-conversion system) to that of the regular device cannula (pre-conversion system). A linear model was used to estimate the difference in average fill volume between the two devices, and a two-sided test at 0.05 significance level was applied for each site and overall. Similarly, two-sided Chi-square tests at 0.05 significance level were employed to compare positivity rates between the two systems for each site and overall. A separate logistic regression was performed to model the effect of average monthly blood fill volume on monthly positivity rate. Prevalence rates for the top 10 organisms identified in the study were calculated based on the bottle positivity rates and the organism prevalence in bottles with available organism identification. All analyses were performed using R (version 4.3.1) (19) along with the “ggplot2” package (20).

## RESULTS

**Fig 1:**
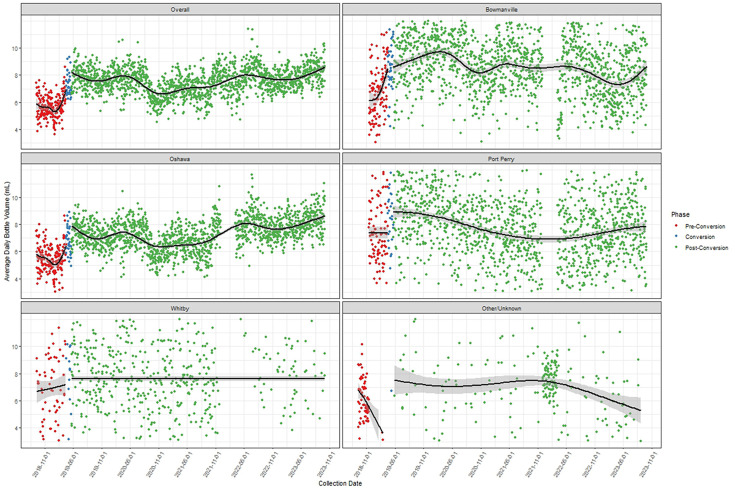
Time series of daily average collection volume by site and overall.

A total of 13,356 pre-conversion and 119,971 post-conversion blood culture blood fill volumes were analyzed across the four hospital sites. Bottles not coded to their specific collection site in the database were included as “unknown” (Table 1). An average of 2,227 bottles were collected monthly; however, two months with lower collection numbers were observed during the study period: 1,205 bottles in September 2018 and 1,510 bottles in June 2022 (Fig. S1). Compared to the average blood culture blood fill volume collected pre-conversion, the observed post-conversion blood fill volume was significantly higher (7.56 mL [95% CI, 7.54, 7.59] versus 5.68 mL [95% CI, 5.62, 5.74], respectively, *P* < 0.001) (Table 1). Blood fill volume also increased significantly at each site, with the average increase ranging from 0.41 mL to 2.06 mL (Table 1). When analyzed as a time series of daily average collection volume by site, important variability was observed over time with volume post-conversion generally higher for sites that had lower pre-conversion volumes (i.e., Oshawa and Bowmanville) (Fig. 1).

**TABLE 1:** Average bottle blood volume and positivity rates pre- and post-conversion^*a*^

Site	Number of bottles (*N*)		Average bottle blood volume mL (95% CI)			Positivity rate % (95% CI)		
	Pre	Post	Pre	Post	*P* value	Pre	Post	*P* value
Bowmanville	1,436	18,510	6.67 (6.47, 6.87)	8.73 (8.67, 8.78)	<0.001	4.7 (3.6, 5.8)	5.9 (5.5, 6.2)	0.068
Oshawa	9,645	82,938	5.41 (5.34, 5.47)	7.30 (7.27, 7.33)	<0.001	6.5 (6.0, 7.0)	7.9 (7.7, 8.0)	<0.001
Port Perry	524	7,991	7.41 (7.11, 7.71)	7.82 (7.74, 7.90)	0.023	11.1 (8.4, 13.8)	6.9 (6.3, 7.4)	<0.001
Whitby	336	2,235	6.54 (6.12, 6.97)	7.54 (7.37, 7.71)	<0.001	23.8 (19.3, 28.4)	17.2 (15.6, 18.7)	0.004
Unknown^*a*^	1,415	8,297	5.73 (5.56, 5.90)	7.29 (7.19, 7.38)	<0.001	6.9 (5.6, 8.2)	8.4 (7.8, 9.0)	0.057
Overall	13,356	119,971	5.68 (5.62, 5.74)	7.56 (7.54, 7.59)	<0.001	7.0 (6.5, 7.4)	7.7 (7.5, 7.8)	0.002

^*a*^ When the specific collection site among the four study locations was not entered in the database, bottles were classified in the “unknown” category.

An increase of 1.88 mL in average blood volume from pre- to post-conversion led to a statistically significant increase in blood culture positivity rates, from 7.0% to 7.7% (*P* = 0.002; OR, 1.11 [95% CI, 1.04–1.19]) (Table 1). Post-conversion, however, positivity rates increased for the two larger sites (Bowmanville, Oshawa) but decreased for the smaller ones (Port Perry, Whitby). Using monthly blood volume data as a predictor for monthly positivity rate in a logistic regression model showed that a 1 mL increase in average volume is associated with a 4.0% relative increase in positivity rate (Fig. S2); however, important variations in monthly positivity rate were observed over time, ranging from approximately 4% to 10% during both pre-conversion and post-conversion periods.

Prevalence rates for the top 10 organisms, based on organism identification data from 4,838/10,164 positive bottles (48%), showed statistically significant increases post-conversion for *Pseudomonas aeruginosa* (0.10% to 0.32%), *coagulase*-negative *Staphylococcus* (0.45% to 0.64%), and *Enterobacter cloacae* complex (0.06% to 0.19%), while a statistically lower prevalence was observed post-conversion for *Streptococcus pneumoniae* (0.32% to 0.14%) (Table S1). Contamination rates remained stable from pre- to post-conversion and consistently stayed below 2% throughout the study period (Fig. S3).

## DISCUSSION

In our study, conversion from regular to ultrathin-wall cannulas for blood collection significantly increased the overall average blood culture fill volume and positivity rate of the assessed blood culture bottles. This concurrent increase in observed blood culture positivity in relation to fill volume aligns with the previously published data by Bouza et al. that reported an approximately 3.5% yield of detection increase for each additional 1 mL of blood cultured (range, 0.6% to 4.7%) (6). Wide variations (4%–10%) in monthly positivity rates in our study indicate that other factors besides fill volume play an important role. In addition to the increase in blood culture collection generally observed during winter months (21), other factors—such as the outcomes of quality improvement programs implemented in healthcare facilities (8, 14, 15), changes in health professionals responsible for blood culture collection at healthcare facilities, variations in the number of patients requiring blood cultures, and local epidemic and global pandemics—may have, either individually or in combination, contributed to these positivity rate variations. When analyzed individually, it was found that the larger sites (Bowmanville and Oshawa) saw their positivity rates increase following conversion, whereas the smaller sites (Port Perry and Whitby) experienced a positivity decrease during the same period. We postulate, given the small sample sizes of these two sites in the pre-conversion period (i.e., 524 bottles for Port Perry and 336 bottles for Whitby compared to 1,436 and 9,645 bottles for Bowmanville and Oshawa, respectively), that higher sample size thresholds for smaller sites may be necessary to assess real-world retrospective data. Notwithstanding these variations, the overall trend of increased fill volume remained consistent in the months following device conversion. This sustained long-term trend in increased fill volume reduces the likelihood that a new-device bias could have impacted the obtained results; moreover, since users were blinded to the conversion, a new-device bias is unlikely to be the source of the observed increase in bottle fill and positivity rate.

### Limitations

The regional scope of our study (four sites of the same healthcare system in Ontario, Canada) may limit the generalizability of the results obtained. Also, from a clinical perspective, it is important to note that the positivity of the blood cultures was determined using instrument level data; consequently, positivity rates obtained in our study may be different from those that would have been observed if electronic patient records had been utilized, as “positivity” may be defined differently at the instrument level compared to the electronic health records. For example, whereas a culture bottle may be positive with an organism, the clinician must correlate the result with the patient’s clinical signs and symptoms. Additionally, due to the sites’ transition in electronic medical record (EMR) system during the study period, not all organism identification in the study samples could be interfaced with the BACTEC instrument, through which the data were obtained. Consequently, organism ID was available for 48% of positive organisms (445/930) pre-conversion and 53% (4,881/9,234) post-conversion. Also, since fill volumes pre- and post-conversion were averaged from blood background signal data in the BACTEC FX instrument at each site, it is possible that the accuracy of the average rates generated for sites with smaller sample sizes may have been impacted. Moreover, for some smaller sites, the limited sample size pre-conversion might have affected the positivity rate for that period, given that a single patient could have multiple positive blood cultures. Of note, false-positive data were not tracked as a study outcome; however, we did not observe major shifts or fluctuations in their incidence throughout the study period. While the sites stated that gage sizes pre- to post-conversion did not change, gage utilization data were not recorded during the study; while unlikely, it is possible that an increased use of 21G needles post-conversion might have influenced the blood fill volume data. Finally, it was not possible to attribute results for the bottles in the “unknown” category to their respective sites, which may have otherwise changed the rates obtained, especially for sites with limited sample sizes.

### Conclusion

Conversion from a regular to an ultrathin-wall cannula blood collection device significantly increased the average blood culture fill volume and positivity rates at the four participating sites and confirmed a positive correlation between increased fill volume and sample positivity. In this context, using ultrathin-wall cannula blood collection devices may support improved detection of positive blood culture samples.

## Data Availability

The data sets/regression code used and/or analyzed during the current study are available from the corresponding author upon reasonable request.

## SUPPLEMENTAL MATERIAL

Fig S1, Fig S2, Fig S3, and Table S1 jcm.00208-25-s0001.pdf Fig S1: Number of bottles collected by month; all sites combined. Fig S2 Positivity rate in relation to monthly blood volume pre- to post-conversion. Fig S3 Contamination rate (%), by sites and quarters, pre- and post-conversion periods.Table S1: Prevalence of the top 10 organisms identified in the study in the pre- and post-conversion periods; data derived from organism prevalence in bottles with available organism identification.

ASM does not own the copyrights to Supplemental Material that may be linked to, or accessed through, an article. The authors have granted ASM a non-exclusive, world-wide license to publish the Supplemental Material files. Please contact the corresponding author directly for reuse.
