# Supplementary material for: Enhancing blood culture volume with ultrathin-wall cannula devices
Source: J Clin Microbiol. 2025 Sep 5;63(10):e00208-25. doi: 10.1128/jcm.00208-25 (PMC12505989; doi:10.1128/jcm.00208-25)
Supplement: Fig S1, Fig S2, Fig S3, and Table S1 — Fig S1: Number of bottles collected by month; all sites combined. Fig S2 Positivity rate in relation to monthly blood volume pre- to post-conversion. Fig S3 Contamination rate (%), by sites and quarters, pre- and post-conversion periods.Table S1: Prevalence of the top 10 organisms identified in the study in the pre- and post-conversion periods; data derived from organism prevalence in bottles with available organism identification. [file jcm.00208-25-s0001.pdf]

SUPPLEMENTAL MATERIAL

Supplemental Figure 1. Number of bottles collected by month; all sites combined.

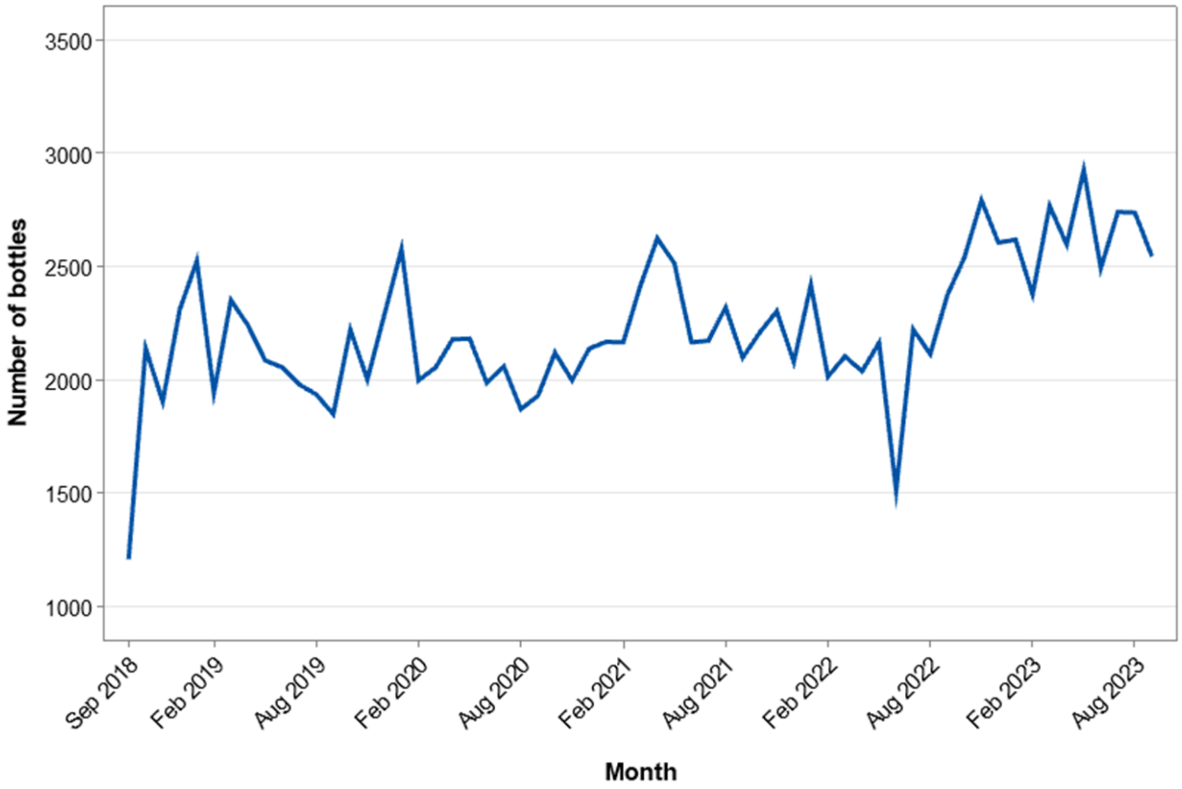

Supplemental Figure 2. Positivity rate in relation to monthly blood volume pre- to post-conversion.

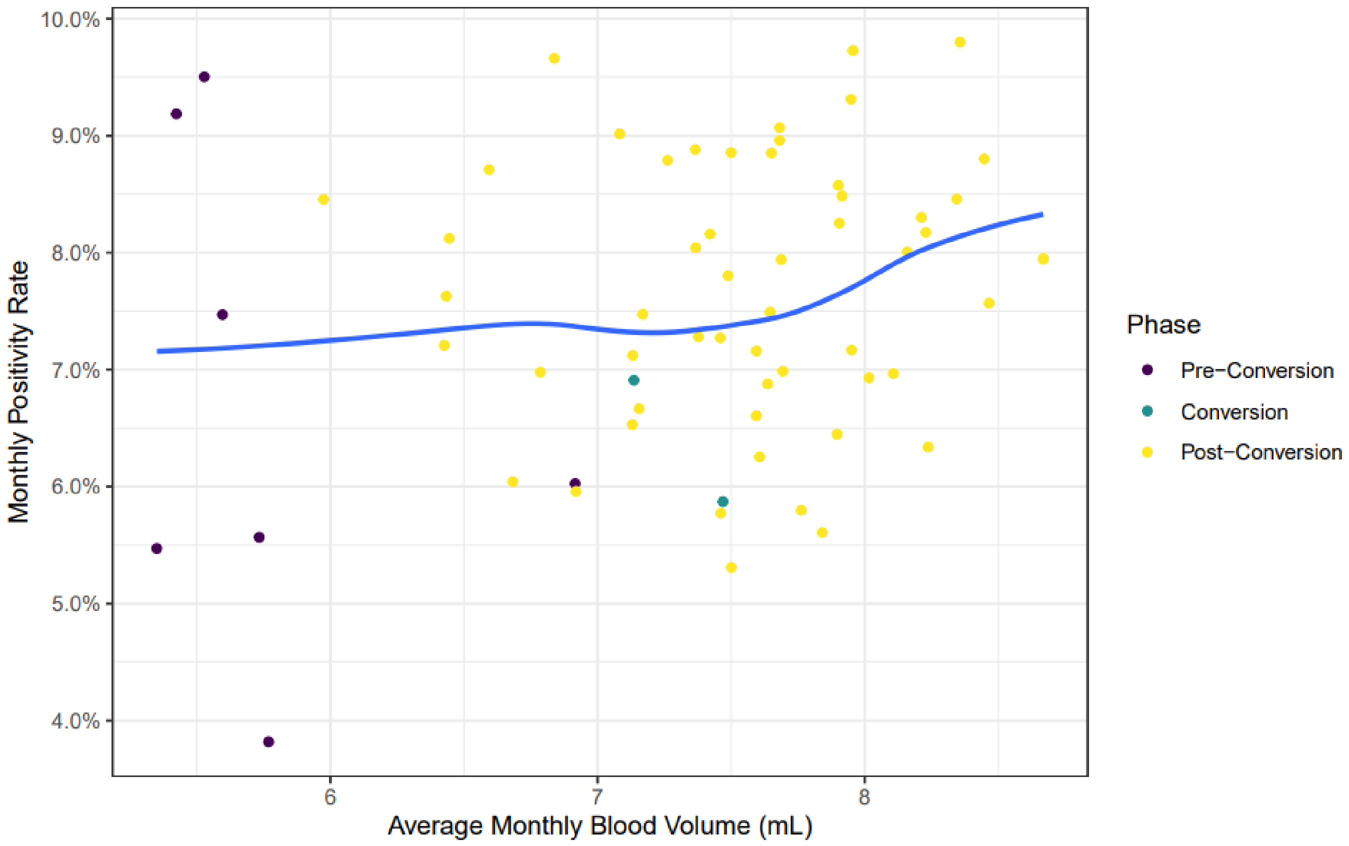

Supplemental Figure 3. Contamination rate (%), by sites and quarters, pre- and post-conversion periods.

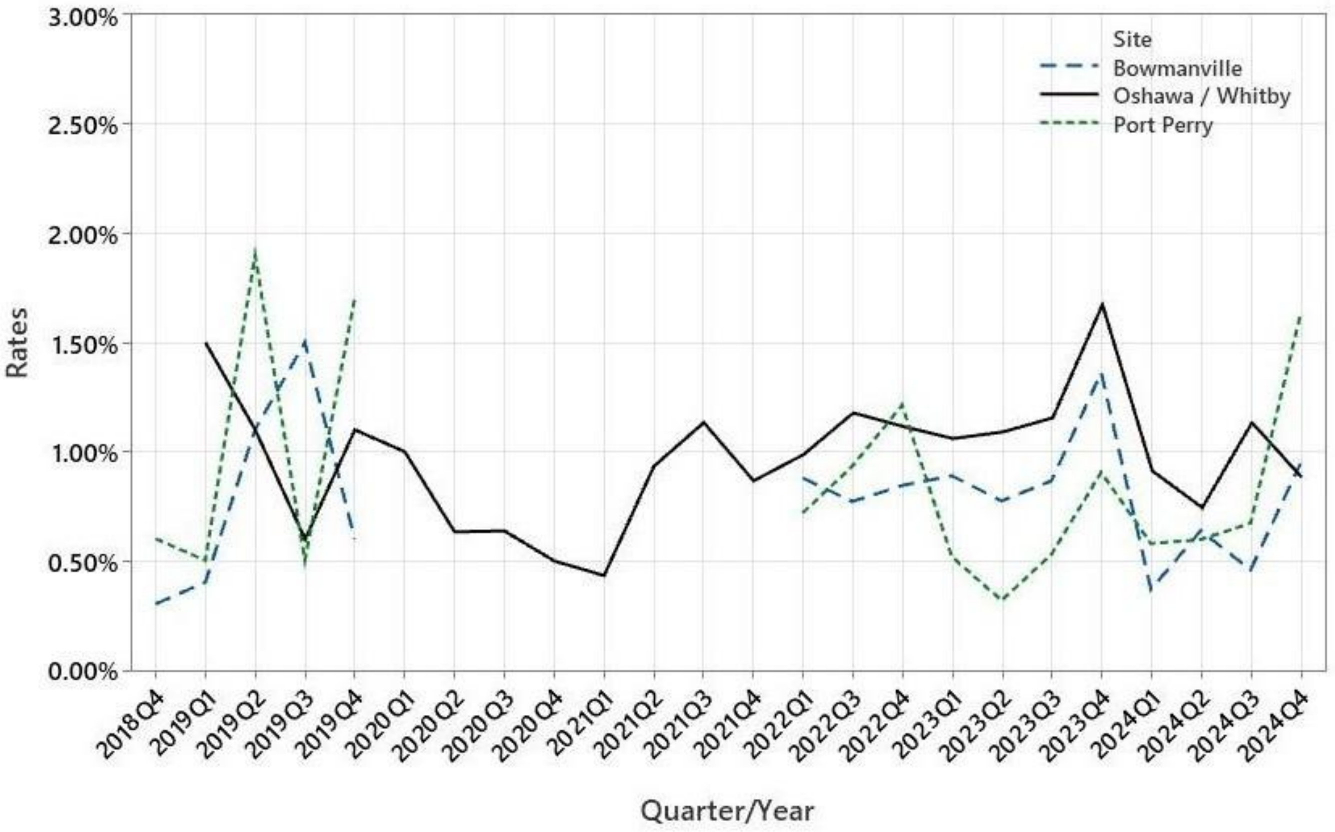

Supplemental Table 1: Prevalence of the top 10 organisms identified in the study in the pre- and post-conversion periods; data derived from organism prevalence in bottles with available organism identification

Organism	Number of organisms	Pre-Conversion Prevalence % (95% CI)	Post-Conversion Prevalence % (95% CI)	Difference % (95% CI)
Escherichia coli	958	1.42 (1.18, 1.69)	1.53 (1.44, 1.61)	7.73 (-9.87, 30.70)
Staphylococcus aureus	872	1.46 (1.23, 1.71)	1.36 (1.28, 1.44)	-7.03 (-19.91, 14.64)
Klebsiella pneumoniae	369	0.53 (0.37, 0.72)	0.59 (0.52, 0.66)	11.84 (-17.73, 63.28)
Staphylococcus epidermidis	309	0.52 (0.36, 0.69)	0.50 (0.45, 0.57)	-2.50 (-28.88, 42.08)
Enterococcus faecalis	278	0.39 (0.25, 0.54)	0.45 (0.39, 0.50)	14.95 (-18.02, 77.59)
Pseudomonas aeruginosa	185	0.10 (0.04, 0.17)	0.32 (0.28, 0.36)	216.69 (85.77, 660.67)*
Streptococcus agalactiae (Strep. group B)	122	0.24 (0.14, 0.37)	0.19 (0.16, 0.22)	-23.21 (-51.54, 40.91)
Other CoNS species (excluding S. epidermidis)	119	0.45 (0.30, 0.61)	0.64 (0.58, 0.71)	44.61 (4.42, 122.23)*
Enterobacter cloacae complex	109	0.06 (0.01, 0.12)	0.19 (0.15, 0.22)	223.29 (47.36, 1318.91)*
Streptococcus pneumoniae	100	0.32 (0.22, 0.47)	0.14 (0.11, 0.17)	-56.34 (-70.98, -35.44)*

Abbreviations: S. epidermidis, staphylococcus epidermidis; CoNS, coagulase-negative staphylococcus
* Represents a statistically significant increase post-conversion
